# Assessing Differences in How the CushingQoL Is Interpreted Across Countries: Comparing Patients From the U.S. and the Netherlands

**DOI:** 10.3389/fendo.2018.00368

**Published:** 2018-07-10

**Authors:** Sonja D. Winter, Sarah Depaoli, Jitske Tiemensma

**Affiliations:** Department of Psychological Sciences, University of California, Merced, Merced, CA, United States

**Keywords:** CushingQoL, quality of life, Cushing's syndrome, measurement invariance, cross-country comparison

## Abstract

**Background:** Cultural factors influence how individuals define, evaluate, and approach their quality of life (QoL). The CushingQoL is a widely used disease-specific questionnaire to assess QoL in patients with Cushing's syndrome. However, there is no information about potential cross-country differences in the way patients interpret the items on the CushingQoL. Thus, the current study examined if the CushingQoL is interpreted in the same way across nationalities.

**Methods:** Patients from the U.S. (*n* = 260) and the Netherlands (*n* = 103) were asked to fill out the CushingQoL and a short demographics survey. Measurement invariance testing was utilized to explore whether or not the patient samples from the U.S. and the Netherlands interpreted items on the CushingQoL in the same way.

**Results:** A two-subscale scoring approach was used for the CushingQoL. Model fit was good for the U.S. sample (e.g., CFI = 0.983; TLI = 0.979), as well as the Dutch sample (e.g., CFI = 0.971; TLI = 0.964). Invariance testing revealed that three of the 12 items on the CushingQoL were interpreted differently across the groups. These items are all related to psychosocial issues (e.g., irritable mood and worrying about one's health). Items assessing physical aspects of QoL did not vary across the U.S. and Dutch samples.

**Conclusions:** Interpreting results from the CushingQoL requires careful consideration of country of residence, as this appears to impact the interpretation of the questionnaire.

## Introduction

Cushing's syndrome (CS) is a rare disease that is characterized by chronic overexposure to elevated cortisol levels (1). CS can have various causes, including: adrenocorticotropic hormone (ACTH) releasing pituitary adenomas, long-term high-dose glucocorticoid steroid use, adrenal tumors, or ectopic tumors (1). Patients with CS experience a number of physical symptoms [e.g., pain, easy bruising, and trouble sleeping (2)] and psychological symptoms (e.g., cognitive impairments, irritability, and impaired quality of life [QoL, (3–5)]. Upon remission, most patients report a reduction in the signs and symptoms of CS, although QoL typically continues to be impaired (5, 6). It is important to accurately assess QoL in patients with CS. The Cushing QoL questionnaire (CushingQoL) was designed specifically for patients with Cushing's syndrome (7). The questionnaire contains 12 items, each with 5 item response categories (depending on the item, the choices are either “Always” to “Never,” or “Very Much” to “Not at All”). The items inquire about different aspects related to QoL that the patient may have experienced in the past 4 weeks. The CushingQoL can either be scored using a total score ranging from 0 to 100 with lower scores indicating a greater impact on QoL [see Webb et al. (7)], or using two different subscales (8). These subscales relate to physical problems and psychosocial issues. Questions in the CushingQoL related to the physical dimension include those such as “I bruise easily,” and questions related to the psychosocial dimension include items such as “I have had to give up my social or leisure activities due to my illness.” Table 1 contains all items in the two subscales, and more information about the structure of this scoring option can be found in Tiemensma et al. (8). For more information on the validity of this questionnaire, see Webb et al. (7). The CushingQoL was developed as a multi-language scale, with papers published using the English version [see e.g., (9)] and Dutch version [see e.g., (10)], among others. However, there are no studies looking into potential cross-country differences in the way patients interpret and answer the items on the CushingQoL. Validating a survey across cultures is an important component of assessing the overall scale performance and interpretation of individual items. Cross-cultural validation studies for surveys have been conducted in a variety of contexts, which include depression (11), personality (12), and the psychological impact of exercise (13), to name a few.

**Table 1:** Items in each subscale of the CushingQoL.

**Psychosocial issues subscale**
2. I have pain that keeps me from leading a normal life 5. I am more irritable, I have sudden mood swings and angry outbursts 6. I have less self-confidence, I feel more insecure 7. I am worried about the changes in my physical appearance due to my illness 8. I feel less like going out or seeing relatives or friends 9. I had to give up my social or leisure activities due to my illness 10. My illness affects my everyday activities such as working or studying 11. It is difficult for me to remember things 12. I am worried about my health in the future
**Physical problems subscale**
1. I have trouble sleeping 3. My wounds take a long time to heal 4. I bruise easily

The U.S. Surgeon General has emphasized the importance of measuring the same construct across nationalities (14). Cultural factors influence how individuals define, evaluate, and approach their health problems. An observed mean difference between groups may be due to true differences between groups on a health outcome. However, it is also possible that these discrepancies are the result of a difference in *interpretation* of individual items across nationalities. There is a statistical process that can be used to assess whether or not different groups are interpreting items in the same way, and this process is referred to as *measurement invariance testing*. If a questionnaire is measurement invariant (MI) across groups, then this result indicates that the items on a questionnaire are interpreted the same across the groups. In other words, the items are tapping into the same underlying construct (e.g., QoL) across groups.

Following the guidelines of the U.S. Surgeon General (14), the aim of the current study is to examine if the CushingQoL is being interpreted in the same way across nationalities, or if there are elements of the items that vary in interpretation.

## Materials and methods

### Subjects

Participants were recruited from the United States (U.S.) and the Netherlands. The study protocol was approved by the University of California, Merced Institutional Review Board. All patients provided digital informed consent in accordance with the Declaration of Helsinki before filling out the survey.

For the U.S. sample, participants were 260 patients who were invited to participate through the Cushing's Support and Research Foundation's (CSRF) listserv and Facebook page. Patients were eligible for the current study if they were over 18 years of age, in remission from CS, and living in the U.S. at the time of completing the survey. Patients were asked to complete the CushingQoL (English version) and a demographics survey. The majority of participants were female (91.1%, *n* = 239). Participants were on average 49.6 years old (*SD* = 13.02).

For the Dutch sample, participants were 103 patients recruited through the Dutch Adrenal Association (NVACP). NVACP members received an email through the NVACP listserv, with a short description of the study and a link to the online survey. Patients were eligible for the current study if they were over 18 years of age, in remission from CS, and living in the Netherlands at the time of completing the survey. Patients were asked to complete the CushingQoL (Dutch version) and a demographics survey. The majority of participants were female (87.4%, *n* = 90). Participants were on average 53.17 years old (*SD* = 12.57).

### Procedure

Patients in both samples received a digital consent form first, which described the nature of the study. After reading and signing the consent form, they were directed to the online survey which included the 12 CushingQoL items as displayed in the original paper-and-pencil version published by Webb et al. (7). The survey was typed in larger font as to mimic the paper-and-pencil version, and all of the items were kept on the same page (as opposed to presenting one item at a time) to exactly mimic the paper-and-pencil version. Upon completion of the survey, patients clicked to the next page, where they received a demographics survey.

### Translation process for converting the CushingQoL to web-based

As mentioned, the CushingQoL was given to patients online as opposed to the original paper-and-pencil version. The translation process for converting the paper-and-pencil version to an online version largely adhered to the guidelines presented by the ISPOR ePRO good research practices task force report (15). In these guidelines, the task force recommends preforming a cognitive debriefing to ensure accurate translation from the original questionnaire format. In the current study, a patient was recruited to participate in a cognitive interview, which was used to assess the validity of the online version of the CushingQoL.

### Statistical analyses

All analyses were conducted in M*plus* version 7.4 (16), and the CushingQoL items were treated as categorical variables given that there were 5 item response choices for each item^1^. Collinearity was evaluated through item correlations on the full sample of valid (i.e., non-missing) responses. No problematic levels of collinearity (i.e., item correlations) were detected. Two questions reflecting the effect of CS on daily activities (items 9 and 10; see Table 1) were highly correlated (*r* = 0.783 for the full sample), but this level of correlation was not severe enough to cause problems in the invariance phase. Therefore, all models were estimated using all CushingQoL items.

To test whether a questionnaire is MI, an analysis called multiple-group confirmatory factor analysis (MGCFA) can be used. MGCFA assesses whether or not items are related to constructs in the same way across the groups. An iterative modeling approach is implemented, where several phases of the model are estimated (17). The first phase examines the situation where the groups are allowed to have entirely different item-level interpretations (i.e., the groups can interpret the construct in entirely different ways, and this drives different response patterns for the items across groups). The subsequent phases of the process iteratively add restrictions in the MGCFA that force certain elements of the model to be the same for both groups. By forcing elements to be the same for both groups, model fit can be examined at each phase of the MI process to determine exactly where the groups are the same (or different) in their interpretation of the items.

The initial estimation of the factor structure followed Tiemensma et al. (8), where two subscales in the CushingQoL were specified. Nine items loaded on a subscale representing the presence of psychosocial issues. The other three items loaded onto a second subscale representing the presence of physical problems (see Table 1).

In the next phase, measurement invariance was evaluated by estimating several multiple-group CFA models. This process was used to compare the U.S. and Dutch samples and assess whether the same subscale structure and model results exist across the groups. Following the guidelines prescribed by Meredith (17), three models were estimated and compared under different levels of invariance (i.e., making the subscale structure in the CFA model increasingly similar for the two groups): (Phase 1) configural invariance, where the subscales are measured by the same items across the groups, but nothing is constrained across groups in the model at this point^2^; (Phase 2) metric invariance, where strength of the relationship between the items and the subscales (i.e., factor loadings) are constrained to be equal across groups^3^; and (Phase 3) scalar invariance, where factor loadings and estimates (i.e., item response thresholds) that are linked to the individual item response categories (e.g., “Always” to “Never”) are held equal across the U.S. and Dutch groups^4^. There is an optional fourth phase of invariance testing. Specifically, if any of the model comparisons indicated a significant worsening of model fit, the option of a partially invariant model would be explored (18). In this case, some (but not all) of the factor loadings or item response thresholds are allowed to vary between the U.S. and Dutch samples. This would indicate that certain questions are interpreted and answered in a different way by U.S. and Dutch participants.

Several model fit indices are reported in the analysis section. The χ^2^ difference test [*p* < 0.05 (19)] was used to compare models at each step of invariance testing. The comparative fit index [CFI; for use in invariance testing, see (20)] and Tucker Lewis fit index [TLI (21)] are fit measures where values closer to 1.0 are considered to represented good fit (as opposed to fit values closer to zero). The root mean square error of approximation [RMSEA (22)] was also used to examine the absolute fit for each model, and values closer to zero represent better fit (compared to values closer to 1). These measures were included because the χ^2^ test is known to be sensitive to a variety of assumption violations, as well as larger sample sizes (23, 24). Thus, it is recommended to use several different methods for assessing fit and examine whether or not they coincide.

## Results

### Patient characteristics—U.S. sample (Table 2)

Clinical characteristics of patients residing in the U.S. are presented in Table 2. Of the 260 patients, 186 (72%) had been diagnosed with Cushing's disease, 73 patients (28%) with Cushing's syndrome, and one patient was unsure of their exact diagnosis. One hundred and eighty-one patients (70%) underwent transsphenoidal surgery, and 20 (8%) patients received postoperative radiotherapy. Fifty (19%) patients underwent unilateral adrenalectomy, and 45 (17%) patients underwent bilateral adrenalectomy. The mean remission duration was 6.97 ± 7.4 years. Ninety-nine (38%) patients reported hypopituitarism. One hundred and nine patients (42%) reported using hydrocortisone, and 46 (18%) patients reported using fludrocortisone.

**Table 2:** Clinical characteristics.

	**Total U.S. sample of patients with Cushing's syndrome (*n* = 260)**	**Total Dutch sample of patients with Cushing's syndrome (*n* = 103)**	***P*-value**
Gender (male/female)	19/239 (2 unknown)	13/90	0.113
Age in years	49.64 (13.0)	53.18 (12.6)	0.020
Educational level (*n*)	No degree: 3 (1%) High school/GED: 36 (14%) Associate's degree: 56 (22%) College degree: 88 (34%) Professional or graduate degree: 77 (30%) (4%)	No degree: 1 (1%) High school/GED: 31 (30%) Associate's degree: 21 (20%) College degree: 37 (36%) Graduate degree: 12 (12%)	<0.001
Diagnosis	Cushing's disease: 186 (72%) Cushing's syndrome: 73 (28%) Unknown: 1 (1%)	Cushing's disease: 63 (61%) Cushing's syndrome: 36 (35%) Unknown: 4 (4%)	0.012
Transsphenoidal surgery, *n* (%)	181 (70%)	63 (61%)	0.122
Adrenal surgery, *n* (%)	Unilateral: 50 (19%) Bilateral: 45 (17%)	Unilateral: 20 (19%) Bilateral: 30 (29%)	0.034
Postoperative radiotherapy, *n* (%)	20 (8%)	22 (21%)	<0.001
Duration of remission in years	6.97 (7.4)	14.26 (12.1)	<0.001
Hypopituitarism, *n* (%)	99 (38%)	47 (46%)	0.186
Hydrocortisone substitution, *n* (%)	109 (42%)	68 (66%)	<0.001
Fludrocortisone, *n* (%)	46 (18%)	29 (28%)	0.026
Physical problems subscale	51.15 (24.3)	47.47 (24.8)	0.198
Psychosocial issues subscale	45.96 (23.5)	46.60 (21.5)	0.811

*Data are mean (SD) or number (%)*.

### Patient characteristics—Dutch sample (Table 2)

Clinical characteristics for the Dutch patient sample are presented in Table 2. Almost two-thirds of the sample was diagnosed with Cushing's disease (61%), and one-third with Cushing's syndrome (35%). Only four (4%) patients were unsure about their exact diagnosis. Sixty-three (61%) patients underwent transsphenoidal surgery, and forty-seven (46%) patients received postoperative radiotherapy. Twenty (19%) patients underwent unilateral adrenalectomy, and 30 (29%) patients underwent bilateral adrenalectomy. The mean duration of remission for this sample was 14.26 ± 24.8 years. Hypopituitarism was reported by 47 (46%) patients. Sixty-eight (66%) patients reported using hydrocortisone and 29 (28%) reported using fludrocortisone.

### Invariance testing (Tables 3, 4)

First, the two-scale factor structure was examined for each group separately. The model fit indices all suggested that the two-scale factor solution reflected the response patterns in the data well for both groups. Fit indices for the U.S. sample were as follows: χ^2^ = 134.69, *p* < 0.001; CFI = 0.983; TLI = 0.979; RMSEA = 0.077 (90% CI = 0.061–0.093). For the Dutch sample, fit indices were as follows: χ^2^ = 102.30, *p* < 0.001; CFI = 0.971; TLI = 0.964; RMSEA = 0.095 (90% CI = 0.067–0.123). Thus, we continued with the multiple-group CFAs to test the different phases of invariance.

**Table 3:** Fit indices for models testing various phases of measurement invariance.

	**χ^2^ (df)**	**Δ χ^2^ (df)**	**CFI**	**TLI**	**RMSEA**
Configural	231.76^*^ (106)		0.981	0.976	0.081 (0.067–095)
Metric	220.26^*^ (116)	14.99 (10) *ns*	0.984	0.982	0.070 (0.056–084)
Scalar	356.23^*^ (162)	167.73^*^ (46)	0.971	0.976	0.081 (0.070–093)
Partial 1	308.85^*^ (158)	107.02^*^ (42)	0.977	0.981	0.073 (0.060–084)
Partial 2	282.84^*^ (154)	74.07^*^ (38)	0.981	0.983	0.068 (0.055–080)
Partial 3	269.65^*^ (150)	57.53^*^ (34)	0.982	0.984	0.066 (0.053–079)

* *p < 0.05; ns, non-significant; Partial 1, Thresholds for item 12 free; Partial 2, Thresholds for item 10 and 12 free; Partial 3, Thresholds for item 5, 10, and 12 free*.

**Table 4:** Illustration of item response patterns for three items.

	**United States**		**Netherlands**	
	**Proportion**	***n***	**Proportion**	***n***
**ITEM 5: I AM MORE IRRITABLE, i HAVE SUDDEN MOOD SWINGS AND ANGRY OUTBURSTS**				
Always	0.054	14	**0.087**	**9**
Often	0.173	45	**0.233**	**24**
Sometimes	0.327	85	**0.369**	**38**
Rarely	**0.373**	**97**	0.243	25
Never	**0.073**	**19**	0.068	7
**ITEM 10: MY ILLNESS AFFECTS MY EVERYDAY ACTIVITIES SUCH AS WORKING OR STUDYING**				
Very Much	0.233	58	**0.282**	**29**
Quite a Bit	0.177	46	**0.291**	**30**
Somewhat	0.227	59	**0.243**	**25**
Very Little	**0.208**	**54**	0.117	12
Not at All	**0.165**	**43**	0.068	7
**ITEM 12: I AM WORRIED ABOUT MY HEALTH IN THE FUTURE**				
Very Much	**0.369**	**96**	0.117	12
Quite a Bit	0.242	63	**0.311**	**32**
Somewhat	0.308	80	**0.359**	**37**
Very Little	0.065	17	**0.175**	**18**
Not at All	0.015	4	**0.039**	**4**

*Bolded values reflect for which group a higher proportion of participants endorsed the answer category*.

The fit indices for each of the phases are reported in Table 3. Phase 1, the configural model, fit the data well. This implies that the way in which the items are related to the two subscales is the same across both groups. However, the item-level interpretations are still allowed to be completely different in this phase.

The second phase, testing metric invariance, did not result in a significant decrease in model fit, as shown by the non-significant χ^2^ difference test. This implies that the strength of the relationship between the items and the subscales is the same across the two groups.

The third phase, testing scalar invariance, did result in a significant decrease in model fit, as shown by the significant χ^2^ difference test. This implies that U.S. and Dutch participants with the same subscale score do not have exactly the same underlying answer pattern on the individual subscale items. Consequently, several partially invariant models (phase 4) were explored.

The fourth phase focused on identifying thresholds (i.e., item response categories) that caused the biggest decrease in model fit. Factor loadings were not considered in these partial invariant models, as phase 2 indicated that they could be held equal across the two groups.

Four partial models were estimated. The first three models examined releasing additional thresholds based on changes in the χ^2^ value, starting with those for item 10, followed by those for item 12, and item 5 (see Table 1)^5^. These three items were all part of the Psychosocial Issues subscale of the CushingQoL. For each of these models, the χ^2^ difference test indicated that the model still fit the data worse than the metric model from phase 2. For the fourth partial model, the added restriction (on a residual variance) did not impact substantive interpretation so the analysis process was concluded.

Thus, the model that best reflected both substantive knowledge and statistical fit to the data only allowed the thresholds of item 5, 10, and 12 to vary across groups. Table 4 illustrates that these three items had different response patterns across the two groups. It is important to note that none of the items on the Physical Problems subscale were allowed to vary, which indicates that for this subscale, participants from both countries did interpret and respond to the items in the same way.

To assess the impact of the noninvariance on the overall composition of the subscales, we compared two versions of the model to see if the U.S. and Dutch participants would differ. The first version assumed all item responses were interpreted the same across groups (i.e., the item thresholds were the same, also called *scalar MI*—phase 3), and the second version allowed the item responses for items 5, 10, and 12 (all from the Psychosocial Issues subscale) to vary across the U.S. and Dutch participants (also called *partial MI*—phase 4) (25). The comparison was specifically to assess whether the subscale means were comparable across the two groups under these modeling conditions. The U.S. and Dutch participants did not differ in their mean subscale scores for the first version (i.e., the scalar model—phase 3) of the model (*B* = 0.014, *SE* = 0.12, *p* = 0.902), or for the second version assessed here (i.e., the partial model—phase 4) of the model (*B* = 0.068, *SE* = 0.12, *p* = 0.561). The MI results indicated that the CushingQoL's Psychosocial Issues subscale does not measure exactly the same construct in U.S. and Dutch samples. However, this final assessment uncovered that the analysis of subscale mean differences across groups led to the same conclusion (i.e., there was no significant mean difference) even if the model (wrongly) assumed that items were interpreted exactly the same across groups (i.e., the scalar model—phase 3). This indicates that the impact of the difference in item interpretation between these groups might be limited in scope.

## Discussion

The aim of the current study was to examine if CushingQoL is being interpreted in the same way across nationalities, or if there are elements of the items that vary in interpretation. Following the U.S. Surgeon General recommendations, it is important to assess whether the same construct (i.e., QoL) is measured across nationalities (14). Measurement invariance testing was utilized to explore whether or not the patient samples from the U.S. and the Netherlands are interpreting items on the CushingQoL in the same way.

The current study found that the U.S. and Dutch groups differed in their interpretation of some of the CushingQoL items. More specifically, they differed in their endorsement of response categories for three items. The Dutch sample was more likely to endorse two of these items (item 5 and 10). For both of these items, Dutch participants were more likely to indicate that they experienced lingering effects of their illness on their daily lives. The U.S. sample was more likely to endorse item 12; they were more likely to express worry about their future health.

These results should be interpreted as exploratory in nature. The procedure for assessing partial MI is data-driven, and as such, the results found here might not replicate in new samples. However, we assessed whether there were actual differences in response pattern between the U.S. and Dutch samples (see Table 4) before allowing certain items to vary between the two groups. This limits the possibility that the findings were due to chance. Cross-validation of the findings found in the current study would provide additional support.

Some of the differences observed could be due to dissimilar sociodemographic and clinical characteristics between the U.S. and Dutch sample. Specifically, the Dutch sample was significantly older, less educated, and reported a longer duration of remission compared to the U.S. sample. In addition, Dutch patients reported a higher incidence of both adrenalectomy and postoperative radiotherapy, and reported a higher usage of Hydrocortisone and Fludrocortisone than the U.S. sample, which is likely due to the differences observed in treatment modalities. These sociodemographic and clinical characteristics could potentially lead to a different interpretation of the items related to lingering effects of Cushing's syndrome on their daily lives. As a result of the self-report nature of this exploration, it is possible that information related to diagnosis, remission status, or treatment (e.g., hormonal supplementations) could be inaccurate if the patient unknowingly misunderstood the information provided by their doctor. In addition, the degree of supplementation of their hormones is unknown.

There are several clinical implications related to these findings. First, researchers conducting a multi-nation study should be mindful to ensure that analyses examining the CushingQoL are separated by nation rather than combining patients across multiple nations. Given the results of the current study, it cannot be assumed that the CushingQoL is being interpreted in the same manner across nations. Thus, it is imperative to separate results by nation when discussing QoL and other research implications. In addition, when clinicians compare CushingQoL scores for individual patients to the literature for score interpretation, it is important that the comparison is made to a body of literature reflecting the country of residence of the patient being examined. Finally, researchers interested in designing interventions surrounding QoL should create the intervention based on individuals residing in the same country where the intervention will be implemented to ensure comparable interpretation of the facets of QoL.

Future studies can examine other nations where the CushingQoL is commonly implemented [e.g., Spain, France, Germany, and Italy (7, 26)] to investigate whether differences in interpretation emerge across groups. It may be that differences will be less substantively impactful between countries close in proximity, with more similar health care systems, and with more overlapping cultures overall. However, a full assessment using the MI process would uncover the exact similarities (and differences) across these nations commonly implementing the CushingQoL. We note that the extent to which we can comment on broader issues related to cross-cultural interpretations of QoL are limited to this patient population. Further research would be needed to assess whether QoL interpretations differ across cultures in a more comprehensive context that spans beyond this patient population.

In summary, the CushingQoL is a valuable tool for assessing the QoL of patients with CS. Interpreting results from this tool requires a careful consideration of country of residence, as this appears to impact the interpretation of the questionnaire.

## Author contributions

All authors wrote and edited the manuscript and approved the final draft. In addition, SW performed the data analysis and interpreted the results, JT conceived the study and collected the data, and SD assisted with data analysis and interpretation.

### Conflict of interest statement

The authors declare that the research was conducted in the absence of any commercial or financial relationships that could be construed as a potential conflict of interest.

## Footnotes

^1^The estimator implemented a weighted least squares-mean and variance adjusted estimator (WLSMV) and theta parameterization.

^2^For identification of the model, the factor means were fixed at zero and the factor variances and residual variances were fixed at one.

^3^This frees a restriction from Phase 1 in that it allows for the estimation of the factor variance in the Dutch group.

^4^The subscale (i.e., factor) mean and variance of the Dutch group are estimated freely.

^5^Modification indices were used to identify thresholds that would lead to the biggest improvement in model fit if they were allowed to differ between the groups. Modification indices reflect the change in χ^2^ that would occur if a certain threshold would be allowed to vary between the two groups. Higher Modification indices indicate a larger decrease in χ^2^ and thus a larger improvement of model fit.

## References

[1] Bista B Beck N. Cushing Syndrome. Indian J Pediatr. (2014) 81:158–64. 10.1007/s12098-013-1203-8 24062268

[2] Sharma ST Nieman LK Feelders RA. Cushing's syndrome: epidemiology and developments in disease management. Clin Epidemiol. (2015) 7:281–93. 10.2147/CLEP.S44336 25945066 PMC4407747

[3] Tiemensma J Biermasz NR Middelkoop HAM van der Mast RC Romijn JA Pereira AM. Increased prevalence of psychopathology and maladaptive personality traits after long-term cure of cushing's disease. J Clin Endocrinol Metab. (2010) 95:E129–41. 10.1210/jc.2010-0512 20660031

[4] Tiemensma J Kokshoorn NE Biermasz NR Keijser B-JSA Wassenaar MJE Middelkoop HAM. Subtle cognitive impairments in patients with long-term cure of cushing's disease. J Clin Endocrinol Metab. (2010) 95:2699–714. 10.1210/jc.2009-2032 20371667

[5] Santos A Crespo I Aulinas A Resmini E Valassi E Webb SM. Quality of life in Cushing's syndrome. Pituitary (2015) 18:195–200. 10.1007/s11102-015-0640-y 25647329

[6] Ragnarsson O Johannsson G. Cushing's syndrome: a structured short- and long-term management plan for patients in remission. Eur J Endocrinol. (2013) 169:R139–52. 10.1530/EJE-13-0534 23985132

[7] Webb SM Badia X Barahona MJ Colao A Strasburger CJ Tabarin A. Evaluation of health-related quality of life in patients with Cushing's syndrome with a new questionnaire. Eur J Endocrinol. (2008) 158:623–30. 10.1530/EJE-07-0762 18426820

[8] Tiemensma J Depaoli S Felt JM. Using subscales when scoring the Cushing's quality of life questionnaire. Eur J Endocrinol. (2016) 174:33–40. 10.1530/EJE-15-0640 26431845

[9] Carluccio A Sundaram NK Chablani S Amrock LG Lambert JK Post KD. Predictors of quality of life in 102 patients with treated Cushing's disease. Clin Endocrinol. (2015) 82:404–11. 10.1111/cen.12521 24931777

[10] Tiemensma J Kaptein AA Pereira AM Smit JWA Romijn JA Biermasz NR. Negative illness perceptions are associated with impaired quality of life in patients after long-term remission of Cushing's syndrome. Eur J Endocrinol. (2011) 165:527–35. 10.1530/EJE-11-0307 21798958

[11] Dere J Watters CA Yu SC-M Bagby RM Ryder AG. Cross-Cultural examination of measurement invariance of the Beck Depression Inventory-II. Psychol Assess. (2015) 27:68–81. 10.1037/pas0000026 25314096

[12] Bowden SC Saklofske DH van de Vijver FJR Sudarshan NJ Eysenck SBG Cross-cultural measurement invariance of the Eysenck Personality Questionnaire across 33 countries. Pers Individ Dif (2016) 103:53–60. 10.1016/j.paid.2016.04.028 [Accessed April 26, 2018].

[13] Griffiths MD Urbán R Demetrovics Z Lichtenstein MB de la Vega R Kun B. A cross-cultural re-evaluation of the Exercise Addiction Inventory (EAI) in five countries. Sport Med - Open (2015) 1:5. 10.1186/s40798-014-0005-5 27747842 PMC4532705

[14] Services USD of H and H Mental Health: Culture, Race, and Ethnicity—A Supplement to Mental Health: A Report of the Surgeon General. Rockville, MD: U.S.: Department of Health and Human Services, Substance Abuse and Mental Health Services Administration, Center for Mental Health Services (2001). 20669516

[15] Coons SJ Gwaltney CJ Hays RD Lundy JJ Sloan JA Revicki DA Lenderking WR Cella D Basch E. Recommendations on Evidence Needed to Support Measurement Equivalence between Electronic and Paper-Based Patient-Reported Outcome (PRO) Measures: ISPOR ePRO Good Research Practices Task Force Report. Outcomes Res (2009) 1098–301509. 10.1111/j.1524-4733.2008.00470.x 19900250

[16] Muthén LK Muthén BO Mplus User's Guide. Seventh Edition. Los Angeles, CA: Muthén & Muthén (1998–2015).

[17] Meredith W Measurement invariance, factor-analysis and factorial invariance. Psychometrika (1993) 58:525–43. 10.1007/Bf02294825

[18] Byrne BM Shavelson RJ Muthén B Testing for the equivalence of factor covariance and mean structures: The issue of partial measurement invariance. Psychol Bull. (1989) 105:456–66. 10.1037/0033-2909.105.3.456

[19] Gregorich SE. Do self-report instruments allow meaningful comparisons across diverse population groups? Testing measurement invariance using the confirmatory factor analysis framework. Med Care (2006) 44:S78–94. 10.1097/01.mlr.0000245454.12228.8f 17060839 PMC1808350

[20] Cheung GW Rensvold RB Evaluating goodness-of-fit indexes for testing measurement invariance. Struct Equ Model A Multidiscip J. (2002) 9:233–55. 10.1207/S15328007SEM0902_5

[21] Tucker LR Lewis C A reliability coefficient for maximum likelihood factor analysis. Psychometrika (1973) 38:1–10. 10.1007/BF02291170

[22] Steiger JH. Structural model evaluation and modification: an interval estimation approach. Multivariate Behav Res. (1990) 25:173–80. 10.1207/s15327906mbr2502_4 26794479

[23] Brannick MT Critical comments on applying covariance structure modeling. J Organ Behav. (1995) 16:201–13. 10.1002/job.4030160303

[24] Kelloway EK Structural equation modelling in perspective. J Organ Behav. (1995) 16:215–24. 10.1002/job.4030160304

[25] Chen FF. What happens if we compare chopsticks with forks? The impact of making inappropriate comparisons in cross-cultural research. J Pers Soc Psychol. (2008) 95:1005–18. 10.1037/a0013193 18954190

[26] Badia X Valassi E Roset M Webb SM. Disease-specific quality of life evaluation and its determinants in Cushing's syndrome: what have we learnt? Pituitary (2014) 17:187–95. 10.1007/s11102-013-0484-2 23564339 PMC3942630

